# Kumada‐Tamao Catalyst‐Transfer Condensation Polymerization of AB_2_ Monomer: Synthesis of Well‐Defined Hyperbranched Poly(thienylene‐phenylene)

**DOI:** 10.1002/marc.202401153

**Published:** 2025-02-02

**Authors:** Yoshihiro Ohta, Toshiki Hirota, Arisa Yamamoto, Tsutomu Yokozawa

**Affiliations:** ^1^ Department of Materials and Life Chemistry Faculty of Engineering Kanagawa University Rokkakubashi, Kanagawa‐ku Yokohama 221‐8686 Japan; ^2^ Department of Applied Chemistry Faculty of Chemistry and Biochemistry Kanagawa University Rokkakubashi, Kanagawaku Yokohama 221‐8686 Japan

**Keywords:** AB_2_ monomer, catalyst‐transfer condensation polymerization, hyperbranched polymer, Kumada‐Tamao coupling

## Abstract

The synthesis of well‐defined hyperbranched aromatic polymer by Kumada‐Tamao catalyst‐transfer condensation polymerization of AB_2_ monomer is investigated. Grignard monomer **2** is generated by treatment of 2‐(3,5‐dibromophenyl)‐3‐hexyl‐5‐iodothiophene (**1**) with 1.0 equivalent of isopropylmagnesium chloride in THF at 0 °C for 1 h and subsequently polymerized with Ni(dppe)Cl_2_ at room temperature for 1 h. The molecular weight of the obtained polymer increases linearly up to ≈30 000 in proportion to the ratio of [consumed **2**] /[Ni(dppe)Cl_2_]_0_ and in proportion to the conversion of **2**, while a narrow molecular weight distribution is maintained (*M*_w_/*M*_n_ ≤ 1.12). The matrix‐assisted laser desorption/ionization time‐of‐flight (MALDI‐TOF) mass spectrum shows almost a single series of peaks due to polymer with hydrogen at one end and bromine at the other, as in the case of Kumada‐Tamao catalyst transfer condensation polymerization of AB monomers. The degree of branching (DB) of the obtained polymers is 0.70–0.75, irrespective of the degree of polymerization. These results indicate that the polymerization of **2** proceeds in a chain‐growth polymerization manner through the intramolecular catalyst transfer mechanism, affording hyperbranched polymer with higher DB than the theoretical DB value of 0.5 in conventional polycondensation of AB_2_ monomers.

## Graphical abstract

Kumada‐Tamao catalyst‐transfer condensation polymerization of AB_2_ monomer in the presence of Ni(dppe)Cl_2_ in THF affords hyperbranched poly(thienylene‐phenylene) with a narrow molecular weight distribution and controlled molecular weight, which is close to the theoretical molecular weight calculated from the ratio of consumed AB_2_ monomer to Ni(dppe)Cl_2_. The degree of branching (DB) of the obtained polymers is 0.70–0.75.

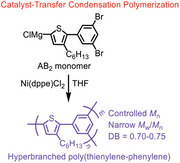

## 1 Introduction

Hyperbranched polymers have received considerable attention due to their unique topological structures and properties.^[^ ^1^, ^2^, ^3^, ^4^, ^5^ ^]^ General synthetic methods for hyperbranched polymers include one‐pot polycondensation or polyaddition of AB_m_ (m ≥ 2) type monomers in a step‐growth polymerization mechanism, though it is difficult to control the molecular weight while achieving a narrow molecular weight distribution. However, the controlled synthesis of hyperbranched polymers has attracted attention because the physical properties of these polymers are dependent on the polymer topology, molecular weight, and molecular weight distribution. In the case of step‐growth polymerization, slow monomer addition^[^ ^6^, ^7^, ^8^, ^9^, ^10^ ^]^ and the use of highly reactive cores^[^ ^11^ ^]^ have been studied. However, the observed molecular weight deviated from the calculated value in some cases even when polymers with a narrow molecular weight distribution were obtained, and the molecular weight distribution became broad when high‐molecular‐weight polymer was synthesized. Accordingly, chain‐growth polymerization was investigated for the synthesis of hyperbranched polymer. Suzuki and Saegusa conducted a pioneer study of the ring‐opening isomerization polymerization of cyclic carbamates as latent AB_2_ monomers from a core molecule in the presence of a Pd catalyst, obtaining hyperbranched polyamine with controlled molecular weight (*M*_n_ = 2000–3000) and a relatively narrow molecular weight distribution (*M*_w_/*M*_n_ = 1.3–1.5).^[^ ^12^, ^13^ ^]^ Gao reported the chain‐growth click polymerization of AB_2_ monomers containing alkynyl and azido groups in the presence of a Cu catalyst, which would be transferred intramolecularly on the triazole group formed by the click reaction.^[^ ^14^, ^15^ ^]^ Yamago examined the synthesis of dendritic hyperbranched vinyl polymers by means of organotellurium‐mediated radical copolymerization (TERP) of vinyl monomer and vinyl telluride.^[^ ^16^, ^17^, ^18^, ^19^, ^20^, ^21^ ^]^ Similar approaches to hyperbranched vinyl polymers by using other radical polymerization methods instead of TERP have also been reported.^[^ ^22^, ^23^, ^24^ ^]^

We established chain‐growth condensation polymerization (CGCP) of AB monomers by utilizing the change of substituent effects between monomer and polymer for the synthesis of well‐defined condensation polymers and intramolecular catalyst transfer for the synthesis of π‐conjugated polymers.^[^ ^25^ ^]^ We applied the former approach to achieve precision synthesis of hyperbranched polyamides by using 5‐(*N*‐alkylamino)benzoic acid ester as an AB_2_ monomer; the molecular weight was controlled up to ≈40 000 (degree of polymerization of 200), while *M*_w_/*M*_n_ remained at 1.1 or less.^[^ ^26^, ^27^ ^]^ In this polymerization, the amide anion monomer, which was generated by the abstraction of a proton from the amino group of the monomer by a base, suppressed self‐polycondensation due to deactivation of the ester moiety through the inductive effect of the strongly electron‐donating amide anion group, and reacted selectively with the initiator and the propagating end bearing no amide anion. However, we have not applied the latter approach, catalyst‐transfer condensation polymerization (CTCP), to the synthesis of well‐defined hyperbranched aromatic polymers. Kim synthesized hyperbranched poly(*m*‐phenylene) with a dispersity of less than 1.5 by means of Suzuki‐Miyaura polycondensation of 3,5‐dibromophenylboronic acid with a Pd(0) catalyst, but addition of more monomer at the end of the polymerization did not increase the molecular weight of the polymer, implying the absence of chain‐growth polymerization behavior.^[^ ^28^ ^]^ Moore investigated Suzuki‐Miyaura polycondensation of *m*‐terphenyl AB_2_ monomer in the presence of Pd(OAc)_2_ and S‐Phos. The molecular weight increased with decreasing amount of the catalyst, but the molecular weight distribution was broadened. The polymerization was proposed to involve a pseudo‐chain‐growth polymerization through intramolecular catalyst transfer, as well as coupling of oligomers to some extent.^[^ ^29^ ^]^

**Scheme 1:**
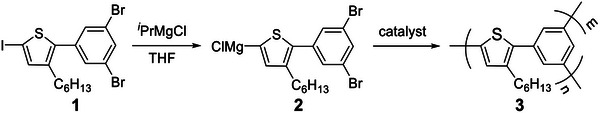
Kumada‐Tamao CTCP of **2**, generated by treatment of **1** with 1.0 equivalent of *^i^* PrMgCl, with a Ni catalyst.

**Scheme 2:**
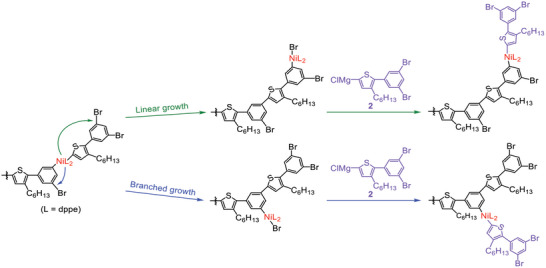
Schematic illustration of linear growth and branched growth during the polymerization of **2** with Ni(dppe)Cl_2_.

We wanted to achieve CTCP of AB_2_ monomer in a more precisely controlled manner, as in the case of CTCP of AB monomers. Since Bielawski reported Kumada‐Tamao CTCP of 1‐bromo‐4‐(5‐chloromagnesio‐2‐thienyl)benzene as a biaromatic AB monomer in the presence of a Ni(II) catalyst,^[^ ^30^ ^]^ and *meta*‐type monomers also underwent Kumada‐Tamao CTCP,^[^ ^31^, ^32^, ^33^ ^]^ we designed Grignard monomer **2**, consisting of thienylene and phenylene groups, for Kumada‐Tamao CTCP leading to a hyperbranched aromatic polymer. In this work, we investigated Kumada‐Tamao polymerization of **2**, generated from the corresponding iodide monomer **1** with isopropylmagnesium chloride, with a Ni catalyst (**Scheme** **1**) and found that the use of Ni(dppe)Cl_2_ (dppe = bis(diphenylphosphino)ethane) enabled the chain‐growth polymerization of **2** via the CTCP mechanism, affording hyperbranched poly(thienylene‐phenylene) with well‐defined molecular weight (*M*_n_ < 30 000), narrow molecular weight distribution (*M*_w_/*M*_n_ ≤ 1.12), and a degree of branching (DB) of 0.70–0.75. The observed high DB indicated that successive substitution of two bromines of the terminal monomer unit with 2 equivalents of **2** would take place through intramolecular Ni catalyst transfer (branched growth) rather than through transfer of the Ni catalyst to the elongated monomer unit (linear growth) (**Scheme** **2**). This catalyst transfer behavior is unique, because CTCP of AB_2_ monomer increases the number of propagating ends as the polymerization proceeds, in contrast to CTCP of AB monomers, which involves only one propagating end during polymerization.

## 2 Results and Discussion

### 2.1 Polymerization Catalysts

To find the optimum catalyst for the polymerization of **2**, three catalysts,^[^ ^34^ ^]^ Ni(dppe)Cl_2_, Ni(dppp)Cl_2_ (dppp = 1,3‐bis(triphenylphosphino)propane), and a Ni complex bearing an *N*‐heterocyclic carbene complex (NHC) as a ligand (Ni(NHC)Cl_2_: [1,3‐bis(2,6‐diisopropylphenyl)imidazol‐2‐ylidene]triphenylphosphine NiCl_2_) were examined because these Ni catalysts are often used for Kumada‐Tamao CTCP (Scheme 1). Thus, the polymerization of **2**, generated by treatment of **1** with 1.0 equivalent of *^i^* PrMgCl, with 10 mol% of Ni(dppe)Cl_2_ was conducted at room temperature for 1 h. The gel permeation chromatogram (GPC) of the obtained crude product showed a narrow peak (*M*_n_ = 3010, *M*_w_/*M*_n_ = 1.11) (Figure S1, Supporting Information). In the polymerization of **2** with Ni(dppp)Cl_2_, GPC showed a slightly broader peak than that in the case of the polymerization with Ni(dppe)Cl_2_ (*M*_n_ = 4210, *M*_w_/*M*_n_ = 1.36) (Figure S2, Supporting Information). However, when Ni(NHC)Cl_2_ was used, the GPC elution curve of the obtained crude product showed multimodal peaks in the lower molecular region (*M*_n_ = 2060, *M*_w_/*M*_n_ = 1.89) (Figure S3, Supporting Information), including a large peak of **7**, formed by quenching of **2** with hydrochloric acid (Scheme S1, Supporting Information), implying that the polymerization did not proceed efficiently. Based on these results, we decided to use Ni(dppe)Cl_2_ for further investigation. The best results obtained by Ni(dppe)Cl_2_ may be accounted for by smooth transmetalation of bulky, hyperbranched polymer‐Ni‐Br end with monomer **2** due to less bulky DPPE ligand.

### 2.2 Polymerization Behavior

**Figure 1:**
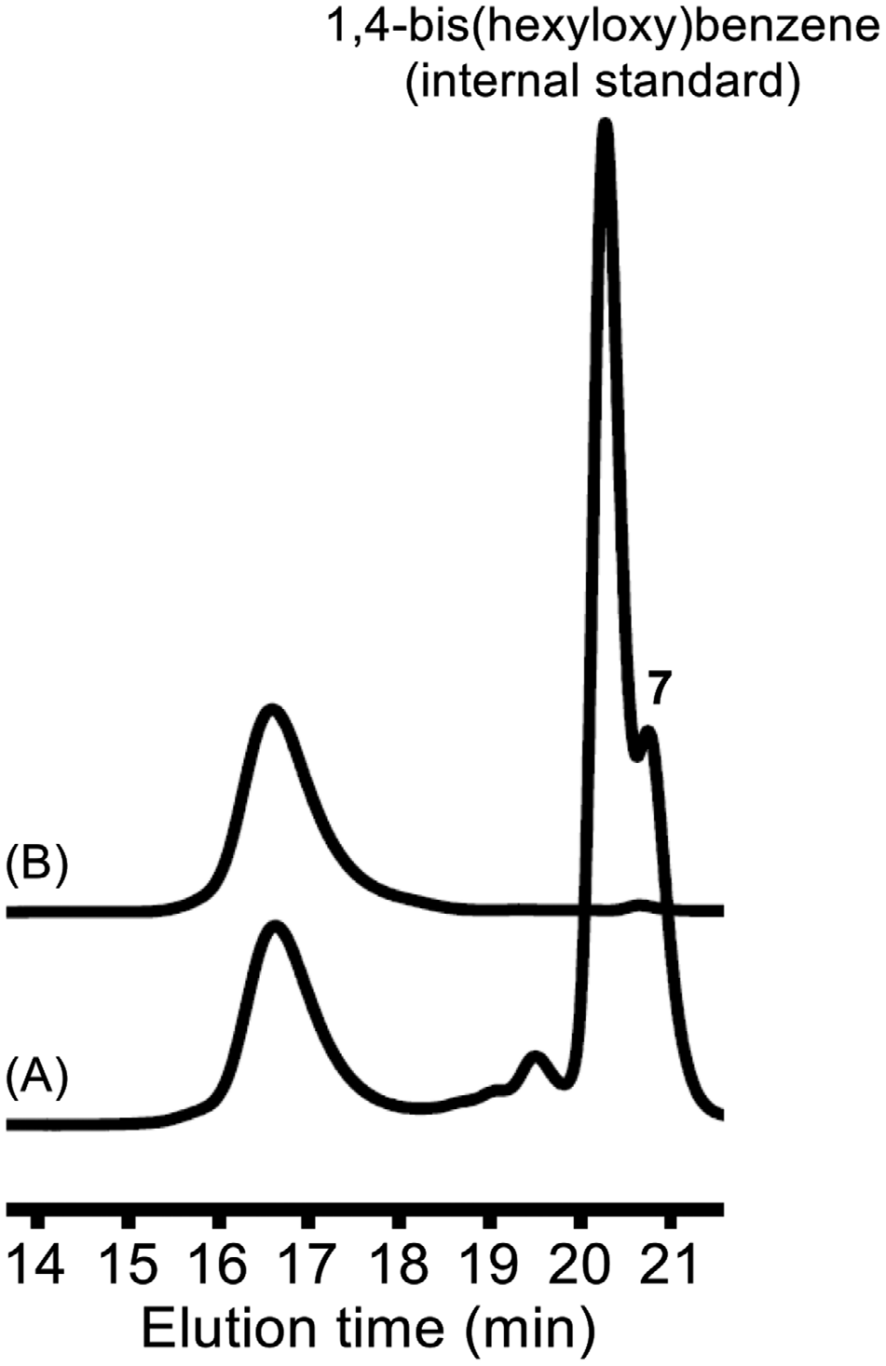
GPC profiles of products obtained by A) the polymerization of **2** with 3.3 mol% of Ni(dppe)Cl_2_ in THF at room temperature, B) followed by purification by preparative HPLC (eluent: CHCl_3_): peak **7** is monomer **2** quenched with hydrochloric acid.

We then investigated the polymerization of **2** with Ni(dppe)Cl_2_ in detail. Iodo monomer presursor **1** was converted quantitatively to Grignard monomer **2** by treatment with 1.0 equivalent of *^i^* PrMgCl at 0 °C for 1 h. The polymerization of **2** with 3.3 mol% of Ni(dppe)Cl_2_ was then carried out at room temperature for 1 h. The conversion of **2**, determined by GC, was 83%. The GPC elution curve of the crude product showed a monomodal narrow peak (*M*_n_ = 6630, *M*_w_/*M*_n_ = 1.10) (**Figure** **1**A). The crude product was purified by preparative HPLC (eluent: chloroform) to remove small molecules, such as internal standard 1,4‐bis(hexyloxy)benzene and **7**, to afford hyperbranched polymer **3**, the GPC peak of which was monomodal (*M*_n_ = 6600, *M*_w_/*M*_n_ = 1.12) (Figure 1B). Since the *M*_n_ value of hyperbranched polymer estimated by GPC is generally lower than the real molecular weight, the absolute molecular weight *M*_w_ was determined with a multiangle laser light scattering (MALLS) detector. To facilitate comparison with the theoretical value (*M*_n_(calcd)) based on the ratio of the consumed **2** to the Ni catalyst, the *M*_n_ value designated as *M*_n_(MALLS) was calculated by division of the *M*_w_ value from MALLS by the *M*_w_/*M*_n_ value from GPC. As a result, the *M*_n_(MALLS) of **3** was 9910, which is quite close to the theoretical value (*M*_n_(calcd) = 8020).

**Figure 2:**
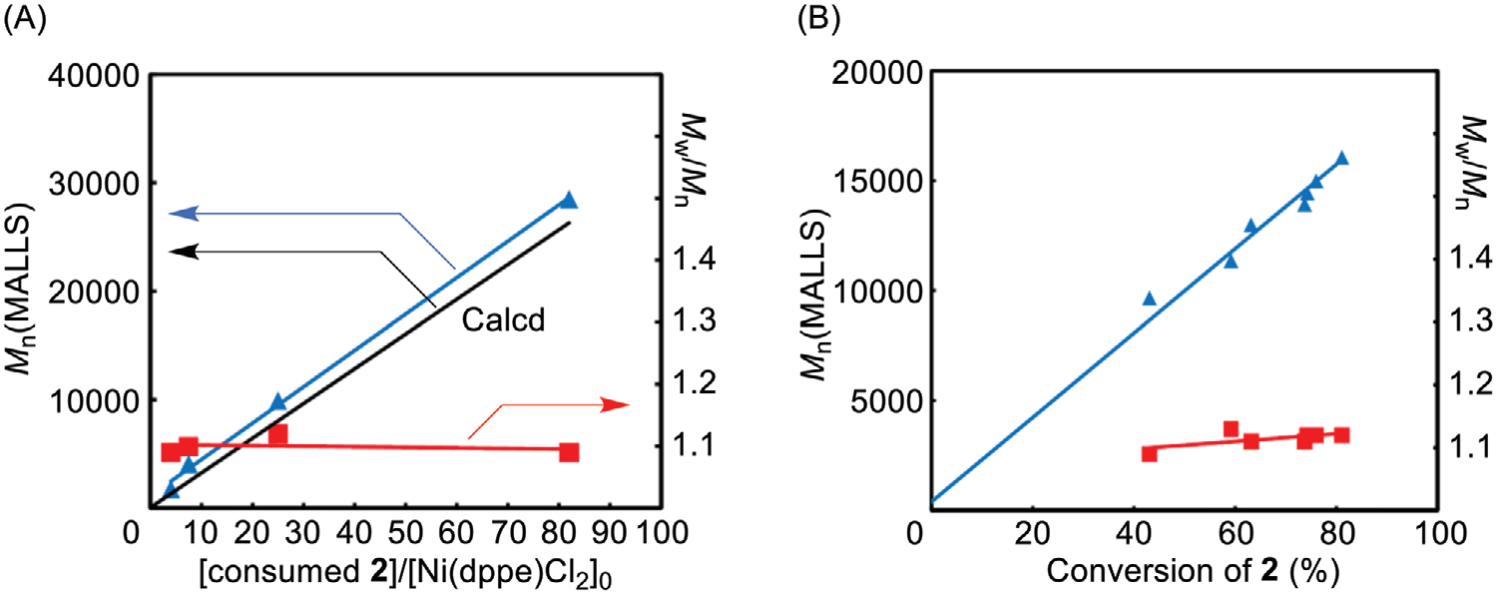
*M*_n_ and *M*_w_/*M*_n_ values of **3** as a function of A) the ratio of consumed **2** to Ni(dppe)Cl_2_ and B) conversion of **2**.

The polymerization was conducted at various feed ratios of **2** to the Ni catalyst to see if the polymerization proceeds in a chain‐growth polymerization manner. The *M*_n_(MALLS) value of **3** was close to the theoretical value calculated from [consumed **2**] /[Ni(dppe)Cl_2_]_0_, and increased linearly in proportion to the ratio of [consumed **2**] /[Ni(dppe)Cl_2_]_0_, while retaining a narrow molecular weight distribution (**Figure** **2**A). It should be noted that polymer with *M*_n_(MALLS) of 28 500 formed a film by casting from a chloroform solution.

Furthermore, we examined the relationship of the molecular weight of **3** to the conversion of **2**. The polymerization was conducted at 0 °C because at room temperature it proceeded too fast to follow the conversion of **2**. The molecular weight, *M*_n_(MALLS) value, was estimated from the *M*_n_(GPC) value by using the relationship between *M*_n_(MALLS) and *M*_n_(GPC), obtained in the previous experiment in Figure 2A (Figure S4, Supporting Information). As a result, the molecular weight of **3** increased in proportion to the conversion of **2**, while a narrow molecular weight distribution was maintained (Figure 2B). The relationships of the molecular weight to ratio of consumed **2** to Ni(dppe)Cl_2_ and monomer conversion indicate that the polymerization of **2** with Ni(dppe)Cl_2_ proceeds in a chain‐growth polymerization manner.

**Figure 3:**
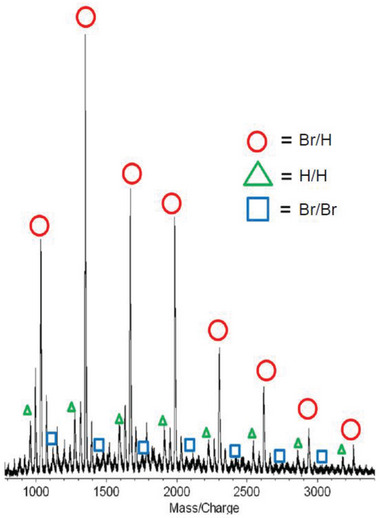
MALDI‐TOF mass spectrum of **3** (*M*_n_(MALLS) = 1760, *M*_w_/*M*_n_ = 1.09).

**Scheme 3:**
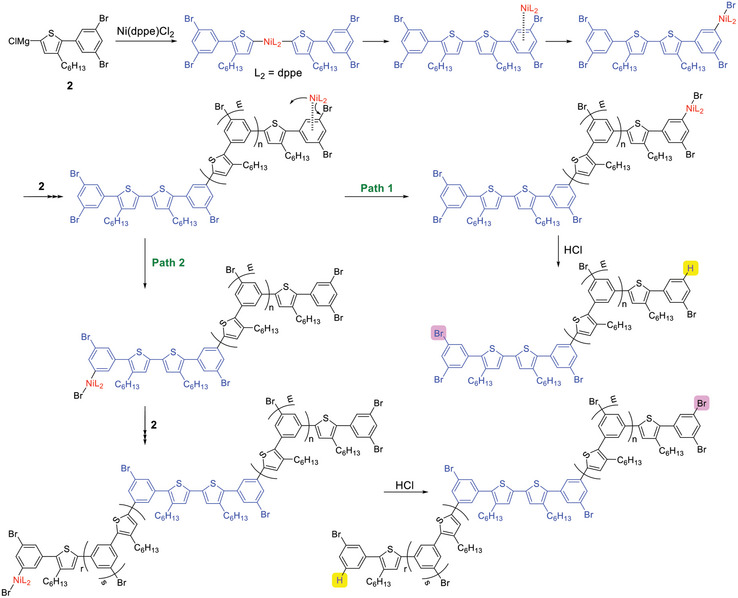
Proposed courses of polymerization in CTCP of **2** with Ni(dppe)Cl_2_: Path 1 is unidirectional growth and Path 2 is bidirectional growth. Yellow H and pink Br are considered to be the end groups, based on peak detection in MALDI‐TOF mass spectra.

The polymer end groups of **3** were analyzed by matrix‐assisted laser desorption/ionization time‐of‐flight (MALDI‐TOF) mass spectrometry. One might think that hyperbranched polymer would have many end groups, in contrast to the case of linear polymer having two end groups. Even hyperbranched polymer, however, has one focal initiation site and one propagating end. The other end groups (bromine in the case of **3**) are regarded as repeat units (hyperbranched polymer with the degree of polymerization of *n* from AB_2_ monomer has one A and (*n* + 1) B ends). Accordingly, the initiation site and the propagating end in hyperbranched polymer can be identified from peaks in the MALDI‐TOF mass spectra. The mass spectrum of **3** (*M*_n_(MALLS) = 1760, *M*_w_/*M*_n_ = 1.09) showed main peaks due to polymer with bromine at one end and hydrogen at the other (designated as Br/H) (**Figure** **3**), as in the case of Kumada‐Tamao CTCP of AB monomers.^[^ ^35^ ^]^ Since the H end is introduced by quenching the polymer‐Ni‐Br complex with hydrochloric acid, the Br end and H end would be the focal point and the propagating end, respectively (**Scheme** **3**). However, if bidirectional growth takes place from the initiation dimer unit (depicted in blue in Scheme 3), as reported in Kamada‐Tamao CTCP of thiophene AB monomer,^[^ ^36^, ^37^, ^38^ ^]^ the Br end is not the focal point but is located in the monomer unit that reacted lastly in the other growth direction (depicted in pink in Scheme 3). It was difficult to determine whether unidirectional growth and/or bidirectional growth occurred in CTCP of **2** from the ^1^H NMR spectrum of **3**.

Minor peaks corresponding to polymer with H/H and Br/Br ends were also observed. The H/H and Br/Br ends would be derived from intermolecular transfer of Ni catalyst. This intermolecular transfer might occur to some extent, because polymer **3** possessed a narrow molecular weight distribution.

### 2.3 Degree of Branching of **3**

**Figure 4:**
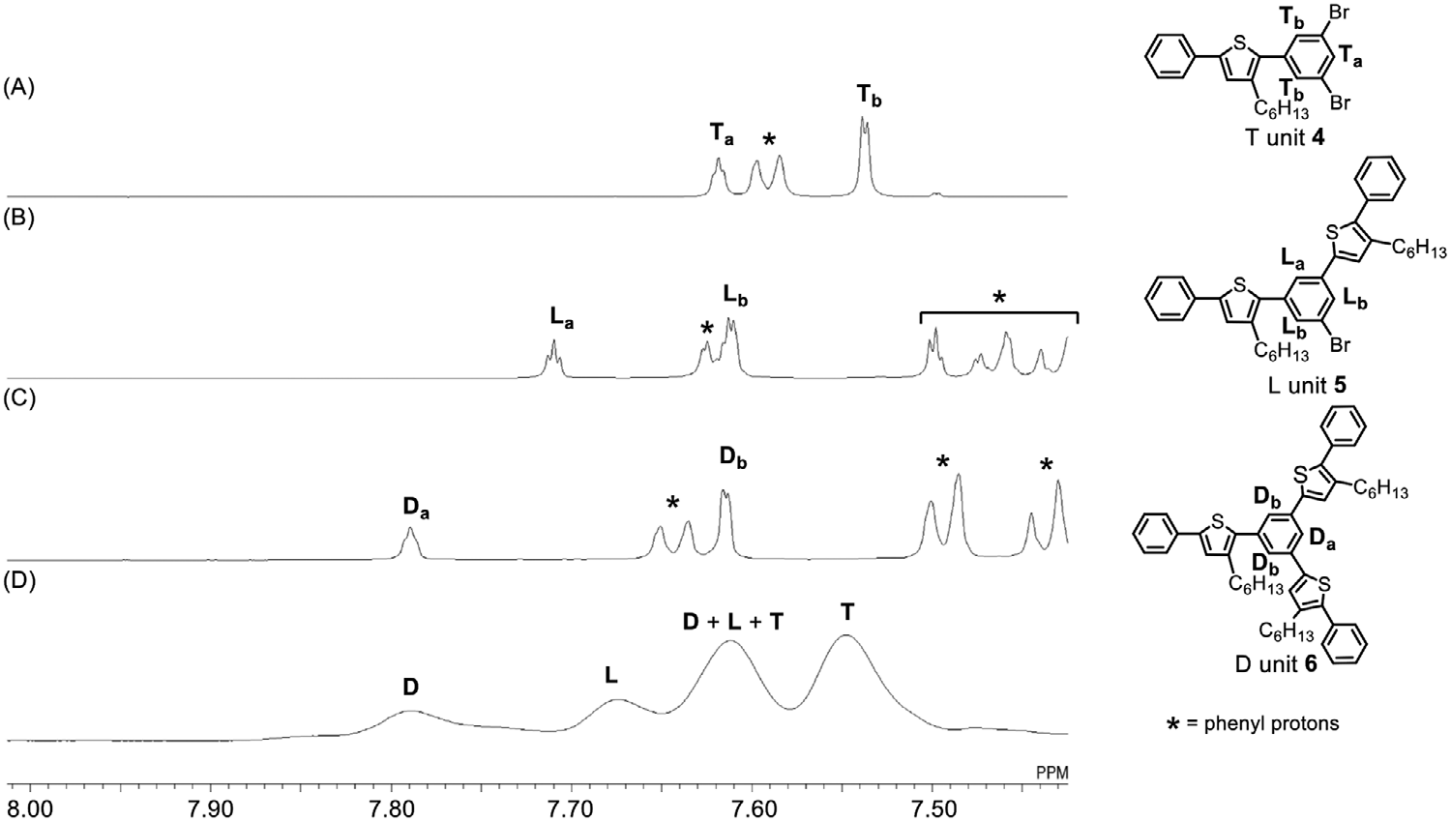
^1^H NMR spectra of the model compounds for A) the terminal unit **4**, B) the linear unit **5**, and C) the dendritic unit **6** of **3**, and D) **3** itself (*M*_n_(MALLS) = 1760, *M*_w_/*M*_n_ = 1.09).

**Figure 5:**
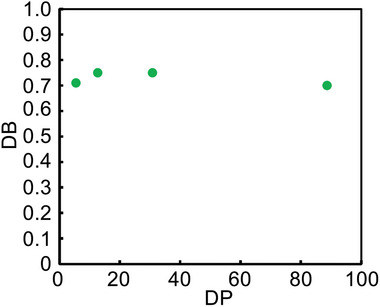
DB values of **3** as a function of DP of **3**.

As mentioned in the introduction, the DB of hyperbranched polymer, obtained by CTCP of AB_2_ monomer, is dependent on the mode of intramolecular catalyst transfer (linear growth versus branched growth) (Scheme 2) and so can provide information on the increase of Br ends with the progress of polymerization in CTCP. We first synthesized model compounds of the terminal (T) unit **4**, the linear (L) unit **5**, and the dendritic (D) unit **6** (Section S5–S7, Supporting Information) and confirmed that the signals of the central benzene ring of **4**–**6** appeared at different chemical shifts in the ^1^H NMR spectra (**Figure** **4**A–C). Based on these ¹H NMR spectra, we assigned four broad signals of **3** as D, L, D + L + T, and T units from low to high magnetic field (Figure 4D). The DBs of **3**, calculated according to Fréchet‘s formula,^[^ ^39^ ^]^ were 0.70–0.75, almost irrespective of the degree of polymerization (DP) of **3** (**Figure** **5**). Since the theoretical DB value of hyperbranched polymer, obtained by conventional polycondensation of AB_2_ monomer, is 0.5,^[^ ^40^ ^]^ the observed DB indicates that **3** possesses well‐branched structures, and it turns out that branched growth occurs more frequently than linear growth in CTCP of **2** with the Ni catalyst. Preferential branched growth might be due to higher electron density of the bromophenyl group substituted with two thienyl groups like the L unit **5** than that of the terminal dibromophenyl group like the T unit **4** in hyperbranched polymer **3** (Scheme 2). Since the Ni catalyst preferentially coordinate to donor aromatics in Kumada‐Tamao CTCP,^[^ ^41^ ^]^ the Ni catalyst might be preferentially transferred to the bromophenyl group rather than the terminal dibromophenyl group after reductive elimination, resulting in branched growth.

## 3 Conclusion

We have demonstrated that Kumada‐Tamao condensation polymerization of Grignard thienylene‐phenylene AB₂ monomer **2** with Ni(dppe)Cl_2_ proceeds in a chain‐growth polymerization manner via the CTCP mechanism, affording well‐defined hyperbranched poly(thienylene‐phenylene) **3**, the DB of which are in the range of 0.70–0.75, indicating that **3** possesses well‐branched structures. Polymerization of **2**, generated by treatment of **1** with 1.0 equivalent of *^i^* PrMgCl, with the Ni catalyst afforded **3**. The molecular weight of **3** increased linearly with increasing [consumed **2**]/[Ni(dppe)Cl_2_]_0_ ratio and with increasing conversion of **2**, while a narrow molecular weight distribution was maintained. The MALDI‐TOF mass spectrum showed almost a single series of peaks due to polymer with H/Br ends, as in the case of Kumada‐Tamao CTCP of AB monomers. Consequently, it turns out that even AB_2_ monomer undergoes Kumada‐Tamao CTCP through not only linear growth but also branched growth, affording well‐defined, well‐branched polymers. Further studies on CTCP of other AB_2_ monomers for the synthesis of well‐defined hyperbranched polymers are in progress.

## Conflict of Interest

The authors declare no conflict of interest.

## Supporting information



Supporting Information

## Data Availability

The data that support the findings of this study are available in the supplementary material of this article.
