# Supplementary material for: Kumada‐Tamao Catalyst‐Transfer Condensation Polymerization of AB2 Monomer: Synthesis of Well‐Defined Hyperbranched Poly(thienylene‐phenylene)
Source: Macromol Rapid Commun. 2025 Feb 2;46(9):2401153. doi: 10.1002/marc.202401153 (PMC12051736; doi:10.1002/marc.202401153)
Supplement: Supplementary file 1 — Supporting Information [file MARC-46-2401153-s001.docx]

Supporting Information

Kumada-Tamao Catalyst-Transfer Condensation Polymerization of AB_2_ Monomer: Synthesis of Well-Defined Hyperbranched Poly(thienylene-phenylene)

Yoshihiro Ohta, Toshiki Hirota, Arisa Yamamoto, and Tsutomu Yokozawa*

**Contents**

1. General

2. Measurements

3. Synthesis of 2-(3,5-dibromophenyl)-3-hexyl-5-iodothiophene (**1**)

4. General procedure for polymerization of **2**

5. Synthesis of T unit **4**

6. Synthesis of L unit **5**

7. Synthesis of D unit **6**

8. Supporting figures

1. **General**

All starting materials were purchased from commercial suppliers (TCI, Aldrich, Wako and Kanto) and used without further purification. Commercially available dehydrated tetrahydrofuran (dry THF, stabilizer-free, Kanto) and dehydrated dichloromethane (dry CH_2_Cl_2_, Kanto) were used as a dry solvent.

1. **Measurements**

^1^H and ^13^C NMR spectra were obtained on a JEOL ECA-600, ECA-500, and ECZ-400R spectrometers. The internal standard for ^1^H NMR spectra in CDCl_3_ was tetramethylsilane (0.00 ppm), the internal standard for ^13^C NMR spectra in CDCl_3_ was the midpoint of CDCl_3_ (77.0 ppm). IR spectra were recorded on a JASCO FT/IR-4600AC. Column chromatography was performed on silica gel (Kieselgel 60, 230-400 mesh, Merck) with a specified solvent. GC was performed on a Shimadzu GC-2010 gas chromatograph equipped with a Shimadzu Rtx-1 column (15 m) and a flame ionization detector (FID). The *M*_n_ and *M*_w_/*M*_n_ values of polymers were measured on a Shodex GPC-101 (eluent: THF, temperature = 40 °C) equipped with Shodex UV-41, Shodex RI-71S, and Wyatt Technology DAWN EOS multiangle laser light scattering (MALLS, Ga-As laser, λ = 690 nm) detectors and two Shodex KF-804-L columns. Calibration was carried out using polystyrene standards. MALDI-TOF mass spectra were recorded on a Shimadzu/Kratos AXIMA-CFR plus by use of laser (λ = 337 nm). *trans*-2-[3-(4-*tert*-Butylphenyl)-2-methyl-2-propenylidene]malononitrile (DCTB) was used as the matrix for the MALDI-TOF mass measurements.

1. **Synthesis of 2-(3,5-dibromophenyl)-3-hexyl-5-iodothiophene (1)**

**Scheme S1**. Synthesis of 2-(3,5-dibromophenyl)-3-hexyl-5-iodothiophene (**1**).

3-1. Synthesis of **7**

All glass apparatus was dried prior to use. Mg metal 0.321 g (13.2 mmol) was placed in a flask, and the flask was heated under reduced pressure. The atmosphere in the flask was replaced with argon and cooled to room temperature. Dry THF (52 mL) was added under a stream of nitrogen, and the atmosphere in the flask was replaced with argon. A solution of 2-bromo-3-hexylthiphene (3.015 g, 12.2 mmol) in dry THF (18 mL) was added under a stream of nitrogen, and the atmosphere in the flask was replaced with argon. The mixture was stirred at room temperature for 1.5 h. The solution was added to a solution of 1,3,5-tribromobenzene (6.027 g, 19.1 mmol) and Pd(PPh_3_)_2_Cl_2_ (0.0868 g, 0.124 mmol) in dry THF (14 mL) under a stream of nitrogen, and the atmosphere in the flask was replaced with argon. The mixture was stirred under reflux for 24 h. The reaction was quenched with ice water after the mixture was cooled to room temperature. The mixture was extracted with AcOEt. The combined organic layers were dried over anhydrous MgSO_4_ and concentrated under reduced pressure. The residue was purified by silica gel column chromatography (hexane) and preparative HPLC (eluent: CHCl_3_) to give 1.557 g of **7** as a light yellow liquid (32%); ^1^H NMR (500 MHz, CDCl_3_, δ): 7.62 (t, *J* = 2.0 Hz, 1H), 7.50 (d, *J* = 2.0 Hz, 2H), 7.26 (d, *J* = 5.4 Hz, 1H), 6.96 (d, *J* = 5.4 Hz, 1H), 2.62 (t, *J* = 7.6 Hz, 2H), 1.59 (quint, *J* = 7.6 Hz, 2H), 1.32-1.21 (m, 6H), 0.87 (t, *J* = 6.9 Hz, 3H); ^13^C NMR (100 MHz, CDCl_3_, δ): 140.1, 138.3, 134.3, 132.6, 130.9, 129.7, 124.9, 122.8, 31.5, 30.8, 29.0, 28.5, 22.6, 14.0; IR (neat): *ν* = 3102, 3061, 2953, 2926, 2855, 1580, 1547, 1449, 1409, 1105, 877, 853, 742 cm^-1^.

3-2. Synthesis of **1**

To a stirred solution of **7** (0.327 g, 0.813 mmol) in dry CH_2_Cl_2_ (5.4 mL) at 0 °C was added iodine (0.114 g, 0.449 mmol) and iodobenzene diacetate (0.172 g, 0.534 mmol) successively, and the mixture was stirred at room temperature for 4 h. Then 10 wt% Na_2_S_2_O_3_ aq. was added, and the mixture was extracted Et_2_O. The combined organic layers were washed with 10% aqueous Na_2_S_2_O_3_, dried over anhydrous MgSO_4_, and concentrated under reduced pressure. The residue was purified by silica gel column chromatography (hexane) to give 0.358 g of **1** as a yellow liquid (83%); ^1^H NMR (500 MHz, CDCl_3_, δ): 7.63 (t, *J* = 1.9 Hz, 1H), 7.43 (d, *J* = 2.0 Hz, 2H), 7.10 (s, 1H), 2.56 (t, *J* = 7.7 Hz, 2H), 1.55 (quint, *J* = 8.0 Hz, 2H), 1.31-1.23 (m, 6H), 0.87 (t, *J* = 7.3 Hz, 3H); ^13^C NMR (100 MHz, CDCl_3_, δ): 142.0, 140.4, 139.4, 137.0, 133.1, 130.7, 122.9, 73.0, 31.5, 30.7, 28.9, 28.1, 22.5, 14.0; IR (neat): *ν* = 3100, 3061, 2953, 2925, 2854, 1579, 1547, 1444, 1408, 1104, 853, 834, 742 cm^-1^.

1. **General procedure for polymerization of 2**

All glass apparatus was dried prior to use. Addition of reagents to a reaction flask and withdrawal of a small aliquot of the reaction mixture for analysis were carried out via a syringe from a three-way stopcock under a stream of nitrogen. A round-bottomed flask equipped with a three-way stopcock was heated under reduced pressure, and then cooled to room temperature under an argon atmosphere. A solution of **1** (0.2186 g, 0.414 mmol) and 1,4-bis(hexyloxy)benzene (0.1185 g, 0.426 mmol), as an internal standard, in dry THF (2.0 mL) was added via a syringe, and the reaction mixture was stirred at 0 °C. 2.0 M Isopropylmagnesium chloride in THF solution (0.20 mL, 0.40 mmol) was added via a syringe, and the mixture was stirred at 0 °C for 1 h. A solution of Ni(dppe)Cl_2_ (0.0427 g, 0.0809 mmol) in dry THF (2.0 mL) was added via a syringe, and the mixture was stirred at room temperature for 1 h. The reaction was quenched with 5 M hydrochloric acid and the mixture was extracted with CHCl_3_. The combined organic layers were washed with water, dried over anhydrous MgSO_4_, and concentrated under reduced pressure. The residue was purified by preparative HPLC (eluent: CHCl_3_) to give 0.0574 g of **3** as a yellow solid (54%).

1. **Synthesis of T unit 4**

**Scheme S2**. Synthesis of T unit **4**.

5-1. Synthesis of **8**

All glass apparatus was dried prior to use. A flask was heated under reduced pressure and the atmosphere in the flask was replaced with argon and cooled to room temperature. 1 M 2,2,6,6-Tetramethylpiperidinylmagnesium chloride lithium chloride complex in THF/toluene (0.60 mL, 0.60 mmol) was added under a stream of nitrogen, and the atmosphere in the flask was replaced with argon. A solution of 3-hexylthiphene (0.0846 g, 0.502 mmol) in dry THF (8.0 mL) was added under a stream of nitrogen, and the atmosphere in the flask was replaced with argon. The mixture was stirred at room temperature for 3 h. Chlorobenzene (0.50 mL, 4.93 mmol) and Ni(dppf)Cl_2_ (0.0071 g, 0.104 mmol) were placed in a flask, and dry THF (14 mL) was added under a stream of nitrogen, and the atmosphere in the flask was replaced with argon. This solution was added to the above solution after stirring for 3 h under a stream of nitrogen, and the atmosphere in the flask was replaced with argon. The mixture was stirred at room temperature for 25 h. The reaction was quenched with 5 M hydrochloric acid, and the mixture was extracted with CHCl_3_. The combined organic layers were washed water, dried over anhydrous Na_2_SO_4_, and concentrated under reduced pressure. The residue was purified by silica gel column chromatography (hexane) to give 0.670 g of **8** as a light yellow liquid (56%); ^1^H NMR (500 MHz, CDCl_3_, δ): 7.58 (d, *J* = 8.2 Hz, 2H), 7.36 (t, *J* = 7.6 Hz, 2H), 7.26 (t, *J* = 6.9 Hz, 1H), 7.15 (s, 1H), 6.86 (s, 1H), 2.61 (t, *J* = 7.6 Hz, 2H), 1.63 (quint, *J* = 7.6 Hz, 2H), 1.39–1.30 (m, 6H), 0.89 (t, *J* = 6.9 Hz, 3H); ^13^C NMR (100 MHz, CDCl_3_, δ): 144.2, 143.9, 134.7, 128.8, 127.2, 125.7, 124.5, 119.4, 31.7, 30.6, 30.4, 29.0, 22.6, 14.1; IR (neat): *ν* = 3082, 3060, 3026, 2954, 2926, 2855, 1599, 1551, 1497, 1454, 1377, 1202, 1072, 838, 759 cm^-1^.

5-2. Synthesis of **9**

*N*-Bromosuccinimide (NBS) (0.156 g, 0.876 mmol) was added into a solution of **8** (0.211 g, 0.863 mmol) in dry THF (2.0 mL), and the mixture was stirred at 0 °C for 3 h. The reaction was quenched with water, and the whole was extracted with EtO_2_. The combined organic layers were washed with 10 wt% Na_2_S_2_O_3_ aq., 10 wt% KOH aq. and water, dried over anhydrous MgSO_4_, and concentrated under reduced pressure to give 0.263 g of **9** as a brown liquid (94%); ^1^H NMR (500 MHz, CDCl_3_, δ): 7.50 (d, *J* = 7.6 Hz, 2H), 7.36 (t, *J* = 7.6 Hz, 2H), 7.28 (t, *J* = 6.2 Hz, 1H), 7.00 (s, 1H), 2.56 (t, *J* = 7.6 Hz, 2H), 1.61 (quint, *J* = 7.6 Hz, 2H), 1.39-1.30 (m, 6H), 0.90 (t, *J* = 6.9 Hz, 3H); ^13^C NMR (100 MHz, CDCl_3_, δ): 143.6, 143.1, 133.9, 128.9, 127.7, 125.4, 124.0, 108.1, 31.6, 29.7, 28.9, 22.6, 14.1; IR (neat): *ν* = 3061, 3025, 2954, 2926, 2855, 1600, 1498, 1453, 1377, 1201, 1072, 1012, 834, 755 cm^-1^.

5-3. Synthesis of T unit **4**

All glass apparatus was dried prior to use. Lithium chloride 0.0267 g (0.630 mmol) was placed in a flask, and the flask was heated under reduced pressure. The atmosphere in the flask was replaced with argon and cooled to room temperature. A solution of **9** (0.1001 g, 0.310 mmol) in dry THF (1.6 mL) was added under a stream of nitrogen, and the atmosphere in the flask was replaced with argon. 2 M Isopropylmagnesium chloride in THF solution (0.16 mL, 0.32 mmol) was added under a stream of nitrogen, and the mixture was stirred at 0 °C for 3 h. A flask was heated under reduced pressure and the atmosphere in the flask was replaced with argon and cooled to room temperature. 1,3,5-Tribrobenzene (0.1478 g, 0.470 mmol) and Pd(PPh_3_)_4_ (0.0188 g, 0.0163 mmol) were charged, and the atmosphere in the flask was replaced with argon. Dry THF (4.3 mL) was added under a stream of nitrogen, and the atmosphere in the flask was replaced with argon. To this solution was added the above solution under a stream of nitrogen after stirring for 3 h, and the mixture was stirred at 70 °C for 24 h. The reaction was quenched with ice water after the mixture was cooled to room temperature. The mixture was extracted with AcOEt. The combined organic layers were dried over anhydrous MgSO_4_ and concentrated under reduced pressure. The residue was purified by silica gel column chromatography (hexane) and preparative HPLC (eluent: CHCl_3_) to give 0.0325 g of **4** as a brown oil (22%); ^1^H NMR (500 MHz, CDCl_3_, δ): 7.62 (t, *J* = 1.7 Hz, 1H), 7.58 (d, *J* = 7.2 Hz, 2H), 7.54 (d, *J* = 1.7 Hz, 2H), 7.38 (t, *J* = 7.6 Hz, 2H), 7.32 (t, *J* = 7.6 Hz, 1H) ,7.19 (s, 1H), 2.63 (t, *J* = 7.5 Hz, 2H), 1.64 (quint, *J* = 7.6 Hz, 2H),1.36-1.25 (m, 6H), 0.88 (t, *J* = 6.5 Hz, 3H); ^13^C NMR (100 MHz, CDCl_3_, δ): 143.5, 141.1, 138.1, 133.9, 133.7, 132.6, 130.7, 128.9, 127.7, 125.7, 125.6, 122.9, 31.6, 30.8, 29.0, 28.8, 22.6, 14.1; IR (neat): *ν* = 3061, 3025, 2953, 2926, 2855, 1599, 1579, 1542, 1499, 1465, 1445, 1417, 1402, 1376, 1104, 1072, 1032, 987, 876, 851, 756 cm^-1^.

1. **Synthesis of L unit 5**

**Scheme S3**. Synthesis of L unit **5**.

6-1. Synthesis of **10**

All glass apparatus was dried prior to use. 2-Bromo-3-hexylthiophene (0.711 g, 2.88 mmol), 2-phenyl-4,4,5,5-tetramethyl-1,3,2-dioxaborolane (0.828 g, 4.06 mmol), CsF (1.078 g, 2.88 mmol), and 18-crown-6 (3.096 g, 11.7 mmol), and P(*t*-Bu)_3_Pd G2 (0.0738 g, 0.144 mmol) were placed in the flask, and the atmosphere in the flask was replaced with argon. Dry THF (10 mL) and distilled water (0.9 mL) were added to the flask under a stream of nitrogen. The mixture was degassed with argon and stirred at room temperature for 2 days. 5 M Hydrochloric acid was added, and the mixture was extracted with CHCl_3_. The combined organic layers were dried over anhydrous MgSO_4_ and concentrated under reduced pressure. The residue was purified by silica gel column chromatography (hexane) to give 0.670 g of **10** as a light yellow liquid (79%); ^1^H NMR (500 MHz, CDCl_3_, δ): 7.44-7.38 (m, 4H), 7.32 (t, *J* = 8.5 Hz, 1H), 7.22 (d, *J* = 5.2 Hz, 1H), 6.98 (d, *J* = 5.2 Hz, 1H), 2.64 (t, *J* = 7.5 Hz, 2H), 1.60 (quint, *J* = 7.6 Hz, 2H), 1.33–1.23 (m, 6H), 0.88 (t, *J* = 6.5 Hz, 3H); ^13^C NMR (100 MHz, CDCl_3_, δ): 138.6, 134.8, 129.5, 129.4, 128.4, 127.2, 123.6, 31.6, 31.0, 29.1, 28.6, 22.6, 14.0; IR (neat): *ν* = 3059, 2955, 2926, 2856, 1599, 1491, 1457, 1444, 1376, 1260, 1092, 1027, 837, 799, 759 cm^-1^.

6-2. Synthesis of L unit **5**

All glass apparatus was dried prior to use. A flask was heated under reduced pressure and the atmosphere in the flask was replaced with argon and cooled to room temperature. 1 M 2,2,6,6-Tetramethylpiperidinylmagnesium chloride lithium chloride complex in THF/toluene solution (0.10 mL, 0.10 mmol) was added under a stream of nitrogen, and the atmosphere in the flask was replaced with argon. A solution of **10** (0.0276 g, 0.113 mmol) in dry THF (0.15 mL) was added under a stream of nitrogen, and the atmosphere in the flask was replaced with argon. The mixture was stirred at room temperature for 3 h. **4** (0.0735 g, 0.154 mmol) and Pd(PPh_3_)_4_ (0.0056 g, 0.00485 mmol) was placed in a flask, and dry THF (0.2 mL) was added under a stream of nitrogen, and the atmosphere in the flask was replaced with argon. This solution was added to the above solution under a stream of nitrogen after stirring for 3 h, and the atmosphere in the flask was replaced with argon. The mixture was stirred at room temperature for 24 h. The reaction was quenched with 1 M hydrochloric acid and the mixture was extracted with AcOEt. The combined organic layers were dried over anhydrous Na_2_SO_4_ and concentrated under reduced pressure. The residue was purified by preparative HPLC (eluent: CHCl_3_) and silica gel column chromatography (hexane) to give 0.0101 g of **5** as a brown oil (14%); ^1^H NMR (500 MHz, CDCl_3_, δ): 7.71 (t, *J* = 1.5 Hz, 1H), 7.64-7.62 (m, 1H), 7.61 (d, *J* = 1.5 Hz, 2H), 7.50-7.33 (m, 7H), 7.29 (t, *J* = 7.5 Hz, 1H), 7.25 (s, 1H), 7.21 (s, 1H), 2.70-2.60 (m, 4H), 1.71-1.59 (m, 4H), 1.40-1.23 (m, 12H), 0.90-0.83 (m, 6H); ^13^C NMR (100 MHz, CDCl_3_, δ): 143.0, 140.7, 139.9, 138.6, 137.0, 136.5, 135.2, 134.3, 134.1, 130.4, 129.2, 128.9, 128.6, 127.59, 127.57, 126.9, 126.6, 125.7, 125.6, 124.8, 123.0, 31.7, 31.6, 31.0, 30.9, 29.2, 29.1, 29.0, 28.8, 22.6, 22.5, 14.1, 14.0; IR (neat): *ν* = 3061, 3024, 2954, 2925, 2855, 1591, 1561, 1499, 1466, 1444, 1377, 1261, 1215, 1098, 1073, 1032, 857, 839, 802, 757 cm^-1^.

1. **Synthesis of D unit 6**

**Scheme S4**. Synthesis of D unit **6**.

7-1. Synthesis of D unit **6**

All glass apparatus was dried prior to use. A flask was heated under reduced pressure and the atmosphere in the flask was replaced with argon and cooled to room temperature. 1 M 2,2,6,6-Tetramethylpiperidinylmagnesium chloride lithium chloride complex in THF/toluene solution (0.10 mL, 0.10 mmol) was added under a stream of nitrogen, and the atmosphere in the flask was replaced with argon. A solution of **10** (0.0480 g, 0.196 mmol) in dry THF (0.30 mL) was added under a stream of nitrogen, and the atmosphere in the flask was replaced with argon. The mixture was stirred at room temperature for 3 h. **4** (0.0480 g, 0.100 mmol) and Ni(dppp)Cl_2_ (0.0052 g, 0.00959 mmol) was placed in a flask, and dry THF (0.2 mL) was added under a stream of nitrogen, and the atmosphere in a flask was replaced with argon. This solution was added to the above solution under a stream of nitrogen after stirring for 3 h, and the atmosphere in the flask was replaced with argon. The mixture was stirred at room temperature for 24 h. The reaction was quenched with 1 M hydrochloric acid and the mixture was extracted with AcOEt. The combined organic layers were dried over anhydrous Na_2_SO_4_ and concentrated under reduced pressure. The residue was purified by preparative HPLC (eluent: CHCl_3_) and silica gel column chromatography (hexane) to give 0.0072 g of **6** as a brown oil (9%); ^1^H NMR (500 MHz, CDCl_3_, δ): 7.79 (t, *J* = 1.5 Hz, 1H), 7.64 (d, *J* = 7.5 Hz, 2H), 7.62 (d, *J* = 1.5 Hz, 2H), 7.49 (d, *J* = 8.0 Hz, 4H), 7.45-7.38 (m, 6H), 7.35 (t, *J* = 7.5 Hz, 2H), 7.30-7.27 (m, 3H), 7.24 (s, 1H), 2.75-2.68 (m, 6H), 1.76-1.64 (m, 6H), 1.42-1.25 (m, 18H), 0.92-0.82 (m, 9H); ^13^C NMR (100 MHz, CDCl_3_, δ): 142.5, 141.3, 140.4, 139.8, 138.0, 136.5, 135.8, 135.3, 134.5, 134.3, 130.9, 129.2, 128.9, 128.5, 127.4, 126.2, 125.8, 125.6, 125.1, 121.5, 31.8, 31.6, 31.3, 31.0, 29.4, 29.2, 29.1, 28.9, 22.7, 22.6, 14.08, 14.06; IR (neat): *ν* = 3060, 3024, 2953, 2926, 2855, 1589, 1548, 1498, 1467, 1444, 1377, 1100, 1073, 1032, 837, 757 cm^-1^.

1. **Supporting figures**

**Figure S1**. GPC profile of the products obtained by the polymerization of **2** with 10 mol% of Ni(dppe)Cl_2_ in THF at room temperature.

**Figure S2**. GPC profile of products obtained by the polymerization of **2** with 10 mol% of Ni(dppp)Cl_2_ in THF at room temperature.

**Figure S3***.* GPC profile of the products obtained by the polymerization of **2** with 10 mol% of Ni(NHC)Cl_2_ in THF at room temperature.

**Figure S4**. *M*_n_(MALLS) values of **3** as a function of *M*_n_(GPC) values of **3**.
